# Vernal keratoconjunctivitis in adults: a narrative review of prevalence, pathogenesis, and management

**DOI:** 10.3389/fopht.2024.1328953

**Published:** 2024-02-15

**Authors:** Antonio Di Zazzo, Angela Y. Zhu, Ken Nischal, Simon S. M. Fung

**Affiliations:** ^1^ Ophthalmology Operative Complex Unit, University Campus Bio-Medico, Rome, Italy; ^2^ Bascom Palmer Eye Institute, University of Miami Miller School of Medicine, Miami, FL, United States; ^3^ Department of Ophthalmology, UPMC Children’s Hospital of Pittsburgh, Pittsburgh, PA, United States; ^4^ Pediatric Ophthalmology, UCLA Stein Eye Institute, Los Angeles, CA, United States

**Keywords:** Vernal keratoconjunctivitis, adult, ocular allergy, epidemiology, pathology

## Abstract

Vernal keratoconjunctivitis (VKC) is a chronic, progressive, allergic ocular surface disorder that can lead to sight-threatening complications. VKC occurs primarily in children and generally resolves about the time of puberty; however, case series and retrospective analyses indicate that approximately 10% of patients with VKC are adults, and that a subset of adult cases develop after puberty. Consequently, two age-related variants of VKC have recently been described: early-onset VKC—which manifests during childhood and persists into adult life—and late-onset disease, which emerges *de novo* after puberty. Although the signs and symptoms of adult and childhood VKC are similar, adult VKC is a long-lasting disease characterized by severe inflammation and increased risk of conjunctival fibrosis, which may place adult patients at higher risk for sight-threatening complications and adverse impacts on daily life. This review discusses the epidemiology, signs, symptoms, immunopathogenesis of adult VKC variants, and highlights current gaps in research and management of patients with this condition.

## 1 Background

Vernal keratoconjunctivitis (VKC) is a chronic, progressive, allergic ocular surface disorder that primarily occurs in children and young adults. Although VKC accounts for less than 1% of ocular diseases in the developed world, prevalence varies widely by region (1, 2). It is most common in warm, humid, windy climates, such as the Mediterranean, Japan, India, and central Africa—where prevalence in excess of 500 cases per 10,000 children have been reported (3)—but is also present in North America, China, Australia, Great Britain, and Sweden (4). The overall prevalence of VKC in Western Europe has been estimated at 3.2 per 10,000 inhabitants (5). In the United States, prevalence among individuals <18 years of age has been estimated at 1.24 per 10,000 people (6).

The signs and symptoms of VKC reflect the conjunctival papillary inflammation and tissue remodeling that are hallmarks of the disease. Patients with VKC experience recurrent inflammatory flares that occur most frequently in the spring and/or summer; however, in some severe cases signs and symptoms may be present throughout the year (7). Severe itching and photophobia are consistent symptoms of VKC (7–9). Left untreated, VKC can lead to sight-threatening complications, including corneal scarring, irregular astigmatism, keratoconus, and limbal stem cell deficiency (2, 8).

VKC is generally recognized as an age-specific disease (2, 10): symptoms typically emerge during the first 10 years of life and either resolve or reduce in severity about the time of puberty (2). However, studies have shown that around 10% of patients with VKC are adults, and many adult cases develop after puberty (11–13). Consequently, two variants of adult VKC have recently been described: early-onset VKC, which begins in childhood and persists into adult life; and late-onset disease, which emerges after puberty (10, 12). In this article, we discuss the pathogenesis of VKC in adults, its impact on patient quality of life, and potential approaches to management.

## 2 Epidemiology and clinical presentation of adult VKC

### 2.1 Epidemiology

The prevalence of VKC in adults has been documented in multiple epidemiologic studies and case series, using a range of age thresholds for defining an “adult”. A retrospective observational case series carried out in Italy by Bonini et al. reported an age range of 3 to 32 years in a cohort of 195 patients with VKC, with an oldest age at symptom onset of 26 years (8). In a 2006 assessment of VKC by age in Padua, Italy (N=406), Leonardi et al. found an incidence of 7.22 per 100,00 in individuals ≤15 years of age versus 0.06 per 100,000 among those >16 years old (14). A 1-year observational study conducted in Kashmir, India (N=212) found that 15% of patients who presented with VKC were >15 years old and 4% were >20 years of age (15).

Studies that have evaluated the age of initial VKC diagnosis or symptom onset suggest that late-onset/post-pubertal VKC accounts for approximately 10% of overall cases and up to 82% of adult cases:

• A 2013 retrospective chart review of patients with VKC in India (N=468) found that 12% were ≥20 years of age at presentation; of these, 29% had adult onset disease (13).
• A 2013 retrospective analysis by Leonardi et al. that included 600 consecutive patients with VKC in Padua, Italy identified 60 (10%) who were adults, 81.6% of whom were diagnosed at >15 years of age (12).
• A 2019 prospective observational study by Di Zazzo et al. compared the pathogenesis, sequelae, and complications of VKC in adult (n=13) versus childhood (n=9) patients at a cornea clinic in India found that 38.5% of adult patients studied had late-onset disease (11).
• More recently, a prospective study conducted at a tertiary care center in India (N=135) found that 11.1% of patients with VKC were adults, and 73.7% of this group had a disease onset >15 years (16).

Unlike early-onset VKC, which is 3 to 4 times more prevalent in males than in females, the male-to-female ratio in late-onset adult disease is more evenly distributed (8, 11, 12, 17). In the aforementioned retrospective case series, Bonini et al. found a male-to-female ratio of 3:2 in patients <20 years of age, which dropped to 1:1 in those older than 20 years of age (8). A similar trend was observed in an earlier study from Israel (N=400), which found that 4.5% of patients of each sex had symptom onset after 20 years of age (18). In the 2006 study in Padua, Italy incidence of VKC among males ≥16 years of age was 0.04 per 100,000 versus 0.08 per 100,000 among females in the same age group (14). Similarly, Leonardi et al’s 2013 retrospective analysis in the same region documented a male-to-female ratio of 2:1 in adult late-onset patients, versus 4:1 in children with VKC (**Table 1**) (12).

**Table 1:** Epidemiologic and clinical characteristics of late-onset adult vs. childhood VKC (12).

Characteristic	Late-Onset Adults	Children	*P* Value
**Male-to-female ratio**	**2:1**	**4:1**	**<0.05**
Type, %			
Tarsal	45	28	NS
Limbal	33	57	NS
Mixed	19	15	NS
Family history of allergy, %	28.6	42.8	0.06
**Corneal ulcer, %**	**4.2**	**18.2**	**0.01**
Asthma, %	10.0	12.0	NS
Rhinitis, %	22.0	26.0	NS
Skin diseases, %	12.0	16.0	NS
**Total serum IgE (KU/L)**	**154 ± 281**	**469 ± 1214**	**0.01**
Eosinophil cationic protein (µg/L)	29 ± 19	34 ± 32	NS

IgE, immunoglobulin E; KU/L, kilounits per liter; NS, nonsignificant. Bold values indicate characteristics with statistically significant difference between groups.

Adapted from Leonardi et al. (12).

### 2.2 Clinical presentation

**Figure 1:**
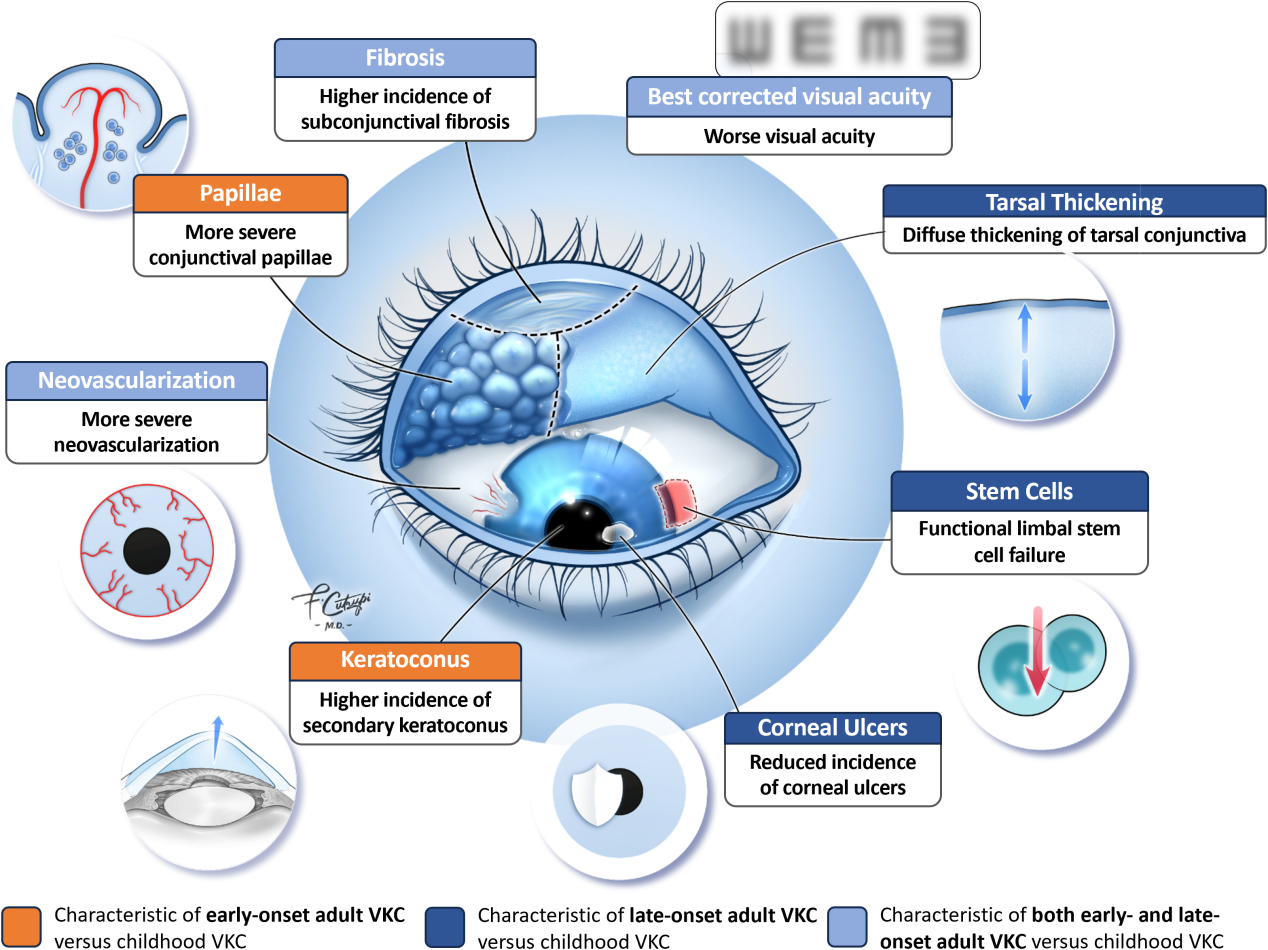
Clinical features of adult VKC vs. childhood VKC. © 2023 Francesco Cutrupi, MD, Ophthalmology, Foundation Campus Bio-Medico University Hospital, Rome, Italy. All rights reserved.

Evidence suggests that the clinical features of VKC are similar across the age spectrum; however, several studies have identified variations in the signs, symptoms, and sequelae of VKC in adults versus children (**Figure 1**). For example, Di Zazzo et al. reported that adult patients with VKC had more severe conjunctival papillae and corneal neovascularization than pediatric patients, regardless of disease onset (11). Looking specifically at adult patients with late-onset disease, Leonardi et al. reported that the tarsal form of VKC appeared as diffuse thickening of the tarsal conjunctiva with subepithelial thickening and fibrosis, but without the prior development of giant papillae observed in children (12). Of note, these patients also were significantly less likely to develop corneal ulcers and corneal involvement than children with VKC (**Table 1**) (12).

It has been suggested that adult patients with VKC are at higher risk of developing severe chronic complications than pediatric patients. Di Zazzo et al. reported that 40% (2/5) of adults with late-onset VKC and 12.5% (1/8) of those with early-onset disease had functional limbal stem cell failure versus 11.1% (1/9) of pediatric patients, and that 25% (2/8) of adults with early-onset VKC had keratoconus versus 22.2% (2/9) of pediatric patients (11). Similarly, in a recent prospective controlled pilot study by Micera et al, when compared to children with VKC (n=9) adult patients with VKC (N=13) were found to have a significantly higher incidence of subconjunctival fibrosis (*P*=0.01) and chronic inflammatory sequelae (*P*=0.001) with significantly worse visual acuity (*P*=0.05) regardless of the time of VKC onset (childhood or late) (19). An earlier retrospective study by Sangwan et al. found that patients who presented with VKC and limbal stem cell deficiency (n=27) were significantly (*P*=0.0005) older (mean age 21.6 years) than patients with VKC alone (mean age 14.7 years) (20). Patients with VKC and limbal stem cell deficiency also had longer disease duration than those with VKC alone (10.0 vs 1.9 years, *P*<0.0001), leading the investigators to postulate that chronic limbal inflammation may adversely affect the stem cell microenvironment, increasing the risk of ocular surface damage (20).

Although the symptoms of VKC in adults are identical to those seen in children, their impact differs. Di Zazzo et al. reported that adult VKC patients (early- or late-onset) had a greater number of hospital visits per year than children with VKC; 69.3% (9/13) of adults visited a hospital more than 3 times a year versus 33.3% (3/9) of children (95% CI: -5.045, 64.022) (11). Leonardi et al. examined the impact of VKC symptoms on adult patients’ quality of life using a modified version of the Quality of Life in Children with Vernal Keratoconjunctivitis (QUICK) questionnaire (12). Adults with late-onset VKC found itching to be the most disturbing symptom experienced, followed by foreign body sensation, burning, and photophobia (12). They experienced symptoms an average of 6 months (± 2.5 months) per year, with varying impacts on work and quality of life. Work productivity decreased by 26% during allergy season, while social activities decreased by 31% (12). Although most patients did not lose workdays—with the exception of those required for ophthalmologic or allergy-related medical visits—patients with more vision-related jobs reported greater declines in productivity (aka presenteeism) (12).

## 3 Immunopathogenesis of VKC in adults

VKC is characterized by a type 1 hypersensitivity reaction that triggers production of immunoglobulin E (IgE) antibodies and culminates in acute symptoms, such as itching, hyperemia, and photophobia (7). In addition, VKC primarily involves a T cell-mediated, lymphocyte T-helper type 2 (Th-2)-driven response characterized by a late-phase allergic reaction with eosinophil infiltration and remodeling of the extracellular matrix (7, 8, 21, 22). Approximately 50% of patients with VKC do not exhibit allergic sensitization (7, 8, 23, 24), and available evidence indicates that adults with late-onset VKC are less likely to have a history of allergy than children with VKC. Di Zazzo et al. found that 23.1% of patients with late-onset VKC had a family history of atopy versus 33.3% of patients with early-onset VKC (11), while Leonardi et al. found that patients with adult-onset disease were less likely to have a family history of allergy than children with VKC (**Table 1**) (12).

Increased androgen levels during puberty may contribute to the resolution of VKC in many patients who develop the disease during childhood (7). Androgen hormones such as dehydroepiandrosterone (DHEA) and DHEA-sulfate (DHEA-S) play key roles in regulating immune function, including suppression of immune reactivity and inflammation (25). As androgen receptors are expressed on a range of immune cells central to the immunopathogenesis of VKC—including T cells, eosinophils, and mast cells—changes in androgen levels have the potential to alter immune cell activation in these patients (25).

In an effort to identify potential differences in androgen hormone levels and inflammatory markers in adult patients with early- and late-onset VKC, Di Zazzo et al. assessed serum levels of DHEA, DHEA-S, and sex hormone binding globulin (SHBG), as well as androgen receptor protein expression and levels of inflammatory marker p65-NFkB on the ocular surface (11). Androgen receptor levels at the ocular surface were higher in adult patients with late-onset and early-onset VKC versus children with VKC (11). Patients with late-onset disease also had increased levels of DHEA and DHEA-S and decreased SHBG versus children with VKC, a result consistent with developmental changes in levels of sex hormones (11). Of note, ocular surface levels of inflammatory marker p65-NFkB were higher in adults with early-onset VKC versus those with late-onset disease (11). Taken together, these findings suggest that differences in local androgen sensitivity may predispose adult patients with VKC to more severe inflammation—with a correspondingly increased risk of sight-threatening complications—than children with the disease (11).

Evidence of heightened inflammation in adult patients was also detected by Leonardi et al, who found increased levels of interleukins IL-1, IL-2, IL-12, interferon gamma (IFN-γ), and granulocyte-macrophage cell stem factor in the tears of patients with the limbal form of late-onset VKC compared to the tarsal form (12). These findings are similar to those of an earlier study by Deligianni et al, who reported a Th-1 type pattern of cytokine production in adult patients with limbal VKC (26).

Most recently, Micera et al. documented increased expression of several key mediators of conjunctival tissue remodeling in adult patients with VKC, providing evidence of a profibrotic phenotype in these patients. Polymerase chain reaction (PCR) analysis found significantly increased expression of smooth muscle alpha-actin (αSMA) and transforming growth factor beta (TGFβ) isoforms in conjunctival imprints of adult patients with early- and late-onset VKC. Similar increases were found in epigenetic modulation of fibrosis gene transcription, particularly in patients with late-onset disease, consistent with a chronic ocular surface inflammatory disease (19).

## 4 Implications for management

The overall principles of management of adult VKC are the same as those for VKC in children, and should be individualized based on the specific manifestations and severity of disease in each patient (10). In cases of mild intermittent disease, maintenance therapy typically consists of topical administration of antihistamines, mast cell stabilizers, or dual-acting agents, coupled with palliative measures such as ocular lubricants (1, 2, 27). More persistent, moderate-to-severe disease may require chronic application of topical immunomodulators (eg, cyclosporine A [CsA], tacrolimus) in addition to short-term pulsed topical steroids as needed (10, 27). Given the higher risk of potentially sight-threatening chronic complications in adult patients with VKC (11, 19, 20), patients with corneal involvement or severe, persistent, or worsening symptoms despite therapy should be referred for specialty treatment, which may include systemic therapy (eg, immunomodulators, biologics) or surgical intervention (eg, debridement of shield ulcers or excision of fibrotic tissue) (27).

Limited information is available on the current treatment patterns of adults with VKC. Di Zazzo et al. found that a majority of adult and pediatric patients received topical immunosuppressives (69.2% and 66.6%, respectively), and that adults with early-onset disease were more likely than late-onset patients to receive topical anti-allergy medications (75% vs 0%). Conversely, adults with late-onset VKC were more likely to be treated with topical steroids (20% vs 0% of early-onset patients) and the duration of topical steroid treatment was longer than in pediatric VKC patients (21.0 vs 15.7 days) (11).

These findings suggest a higher level of inflammation in adults with VKC that may require more aggressive intervention than childhood disease. For example, Di Zazzo et al. noted an increased need for topical steroids and immunosuppressive drugs in adult patients with active VKC (10). However, long-term use of topical corticosteroids and the potential for inappropriate use may increase the risk of vision-threatening complications, including glaucoma and cataracts (28). Di Zazzo et al. reported a high incidence of steroid-induced cataract and glaucoma in their adult cohort, which they noted could be “a reflection of severe disease requiring frequent topical corticosteroid eye drops to treat inflammation” (11).

When treating children with VKC, ophthalmologists have been advised to consider steroid-sparing agents and to monitor children closely for glaucoma when topical steroids are prescribed, to prevent unnecessary blindness (27–29). This same caution should be applied to adults with VKC, who suffer from a potentially lifelong inflammatory disease. Leonardi et al. noted that although adequate treatment was effective at controlling symptoms in adult patients, few achieved complete remission (12). This finding underscores the importance of regular follow-up and monitoring of adult patients to assess treatment efficacy and adjust the management plan as needed.

## 5 Discussion

Current literature suggests that there are differences in the sex distribution, signs, symptoms, and pathogenesis of VKC in adults versus VKC in children. While early-onset and late-onset adult VKC are often discussed together, there may also be differences between these two variants, although the limited number of patients evaluated to date precludes drawing firm conclusions.

A key limitation of the data presented in this review is the small number of patients evaluated in studies of VKC in adults. This is not unexpected, however, given that the prevalence of VKC is about 1% in the population and adults comprise approximately 10% of patients with this disease. Information about adult VKC is also limited by the fact that follow-up for these patients has generally not extended beyond 30 years of age (12). As a result, it is unclear whether adult VKC, like childhood VKC, resolves with advancing age. Leonardi et al. found that VKC persisted in some adult patients for >6 years and resolved completely in only one patient over this period (12). The longer duration of disease, potentially high level of inflammation, and adverse effects on work and social activities reported in adults with VKC suggest that these patients may require significant social support and careful patient monitoring to ensure treatment adherence (9, 12).

Additional research is needed on the hormonal patterns, immunological profiles, and course of symptoms over time in adult patients with VKC. Improved understanding of the immunologic underpinnings of early- and late-onset disease can help inform treatment decisions and identify appropriate steroid-sparing treatment regimens for management of this chronic inflammatory condition. In addition to larger-scale prospective and retrospective studies, meta-analyses of data from existing studies that have included adult patients can help address the many questions that remain about VKC in adults.

## Author contributions

AD: Conceptualization, Visualization, Writing – original draft, Writing – review & editing. AZ: Conceptualization, Writing – original draft, Writing – review & editing. KN: Conceptualization, Writing – original draft, Writing – review & editing. SF: Conceptualization, Writing – original draft, Writing – review & editing.
